# Occupational and Environmental Health Effects of Nanomaterials

**DOI:** 10.1155/2015/789312

**Published:** 2015-06-10

**Authors:** Il Je Yu, Mary Gulumian, Sehyun Shin, Tae Hyun Yoon, Vladimir Murashov

**Affiliations:** ^1^Institute of Nanoproduct Safety Research, Hoseo University, Asan 336-795, Republic of Korea; ^2^Toxicology Department, National Institute for Occupational Health, Johannesburg 2001, South Africa; ^3^Haematology and Molecular Medicine Department, The University of the Witwatersrand, 25 Hospital Street, Constitution Hill, Johannesburg 2001, South Africa; ^4^Green Nanotechnology Center, School of Mechanical Engineering, Korea University, Seoul 136-701, Republic of Korea; ^5^Nanoscale Characterization & Environmental Chemistry Laboratory, Department of Chemistry, Hanyang University, Seoul 133-791, Republic of Korea; ^6^Department of Health and Human Services, National Institute for Occupational Safety and Health, 395 E Street, SW, Patriots Plaza 1, Suite 9200, Washington, DC 20201, USA

With the recent advancements in nanosciences and nanotechnologies, a large number of novel nanomaterials have been introduced in our everyday life. However, as the potential hazards of these novel nanomaterials have not yet been fully understood, concerns on their occupational and environmental health effects are mounting. In fact, recent scientific studies suggested that at least some nanoparticles actively interact with biological tissues and cause toxicological effects on the experimental animals exposed to these materials. Current research also indicates that the toxicity of manufactured nanomaterials will depend on their physical and chemical properties including their chemical composition, core size, morphology, agglomeration state, surface area, and surface charge. However, so far, quantitative understanding on the relationships between their physicochemical properties, biological toxicities, and human and environmental health effects is lacking. It is therefore imperative to have a clearer understanding of such a relationship and hence the aim of this special issue is to discuss occupational and environmental health effects of nanomaterials in relation to their various physicochemical properties.

Several authors participated in this special issue to present their understanding on occupational and environmental health effects of nanomaterials. “Three-Day Continuous Exposure Monitoring of CNT Manufacturing Workplaces” by J. H. Lee et al. and “Workplace Exposure to Titanium Dioxide Nanopowder Released from a Bag Filter System” by J. H. Ji et al. are related to occupational safety and health of nanomaterial and deal with exposure assessment in the workplaces producing or handling manufactured nanomaterials. The authors of these two papers actively monitored exposure situations in the workplaces where nanomaterial manufacturing and handling were conducted and presented workers' exposure situation and exposure mitigation strategies. “Aquatic Toxicity Comparison of Silver Nanoparticles and Silver Nanowires” by E. K. Sohn et al. and “Multiwall Carbon Nanotube-Induced Apoptosis and Antioxidant Gene Expression in the Gills, Liver, and Intestine of* Oryzias latipes*” by J. W. Lee et al. are dealing with aquatic toxicity of one-dimensional form of nanomaterial such as wire or fiber form. E. K. Sohn et al. compared aquatic toxicity of particular forms with wire forms of nanomaterials and J. W. Lee et al. found that the gills were more sensitive to MWCNT toxicity than the other organs with gender difference and caused apoptosis with relevant gene expressions increasing caspases, while reducing expression of catalase and GST genes. Y.-H. Luo et al. studied immunotoxicity of metal-based nanoparticles showing different toxicokinetics from conventional bulk nonnanoscale materials depending on their physicochemical properties. In the paper entitled “Metal-Based Nanoparticles and the Immune System: Activation, Inflammation, and Potential Applications,” a discussion is presented on nanoparticle and innate immunity and effect of nanoparticle exposure on toll-like receptor signaling and on their role in innate immune system and effect of nanoparticle exposure on adaptive immunity.

Currently, there is limited evidence on human hazards due to nanomaterial exposure. This is partly due to the very few numbers of workers or consumers exposed to nanomaterial as well as limited epidemiological or biomonitoring tools to evaluate human health hazards caused by nanomaterial exposure. L. Čábalová et al. studied micro- and nanosized particles using scanning electron microscopy and Raman microscopy in nasal mucosa from patients with chronic hypertrophic rhinosinusitis and obtained the mucosa of the inferior nasal turbinates, which are the deposition site of inhaled particles and the first barrier filtering the inhaled air. Their pilot study suggested possibility of quantification of distribution of micro- and nanosized particles in tissue samples. Their results give some promising methods by which to investigate nanoparticle exposure in human population. Nanomaterial measurement and characterization method using superresolution stimulated emission depletion (STED) microscopy presented a new method in the evaluation of nanoparticle interaction mechanisms on a cellular level. They demonstrated quantitative estimates of the number of nanoparticles administered, delivered, and internalized by epithelial cells which can be extracted from 3D STED image stacks of entire cells via image processing. This method would provide understanding on essential knowledge for risk assessment and safe design approaches in nanotechnology.

